# Telemedicine for headache follow-up: feasibility, clinical outcomes, and patient-reported experience from a prospective study

**DOI:** 10.3389/fneur.2025.1706864

**Published:** 2026-01-09

**Authors:** Roberta Grasso, Carlo Avolio, Ciro Mundi, Giuseppe Raunich

**Affiliations:** 1 Neurology Unit, Azienda Ospedaliero-Universitaria di Foggia, Foggia, Italy; 2 Department of Neurosciences, University of Foggia, Foggia, Italy; 3 Computer Engineer, Data Analyst, Potenza, Italy

**Keywords:** telemedicine, headache, teleconsultation, patient satisfaction, neurological care, follow-up

## Abstract

**Background:**

Telemedicine has emerged as a promising platform in neurology, particularly for facilitating teleconsultations. However, its role in headache management remains largely unexamined, requiring further investigation to assess patient satisfaction, implementation feasibility, and perceived therapeutic benefits.

**Methods:**

We conducted a prospective observational study at the Headache Outpatient Clinic of the A. O. U. of Foggia, enrolling 45 patients with primary headache. Each participant completed two virtual teleconsultations (4–8 weeks apart) using the PHASE platform. After each visit, patients completed a validated 20-item questionnaire (Cronbach’s *α* = 0.92) assessing usability, communication quality, and perceived benefits. Statistical analyses included descriptive statistics, inferential tests, correlation analyses, and effect-size estimates.

**Results:**

All patients completed both teleconsultations. Overall satisfaction was very high (mean >9/10 for both visits), with more than 90% of participants reporting a positive experience. Ease of use reached an average of 6/7, showing significant improvement from T1 to T2. Over 85% of participants reported tangible savings in both time and costs. Willingness to repeat the teleconsultations was also high (~6.7/7) with further increases observed at the second follow-up. Correlation analyses revealed strong associations between patient satisfaction, time savings, and willingness to continue using telemedicine. Effect-size estimates consistently confirmed positive perceptions, suggesting a ceiling effect. Reported limitations were minor and related mainly to occasional technical issues and the inability to perform a full neurological examination.

**Conclusion:**

A structured telemedicine protocol for headache follow-up proved feasibility, effectiveness, and high patient acceptability. Teleconsultations enhanced the perceived quality of care and optimized efficient resource utilization, supporting their integration into routine neurological follow-up. Further validation through larger multicenter studies is needed to confirm these findings and expand the available evidence.

## Background

Headache is one of the most common and disabling neurological disorders, imposing a substantial socioeconomic burden. Migraine affects approximately 14% of the global population, making it one of the most prevalent neurological conditions (1). It is the second leading cause of neurological disability and a major contributor to years lived with disability worldwide (2, 3). In Italy, chronic primary headache has been formally recognized as a social disease, reflecting its considerable impact on quality of life and healthcare resource utilization (4).

Headache management requires continuous monitoring and regular follow-up visits to adjust treatment and ensure adherence. However, access to specialist care is often limited by prolonged waiting times, logistical challenges, and systemic organizational barriers. These factors highlight the need for innovative and more accessible models of care delivery.

Telemedicine, defined as the remote delivery of healthcare services through digital technologies, expanded rapidly during the COVID-19 pandemic, becoming a key tool for ensuring continuity of care in neurology (5). Evidence supports its usefulness in various neurological conditions, including stroke, epilepsy, multiple sclerosis, and Parkinson’s disease. In the context of headache management, teleconsultations have been associated with reduced waiting times, low indirect costs, and high levels of patient satisfaction (6, 7).

Despite these advantages, several challenges remain, including the limitations of remotely conducting neurological physical examinations, the need for adequate technological infrastructure, and the risk of digital literacy inequalities. Although telemedicine has been widely discussed as a strategic tool for neurological care, its implementation in real-world headache practice in Italy remains limited (8). Although many headache centers adopted telemedicine during and after the COVID-19 emergency, only a minority have implemented structured protocols or systematically assessed clinical feasibility, patient experience, and potential barriers. As a result, evidence on the practical challenges, limitations, and benefits of telemedicine in Italian headache care remains sparse and is largely derived from theoretical discussions or emergency-driven initiatives (9).

This study aims to address existing gaps by prospectively evaluating a standardized telemedicine follow-up pathway using validated patient-reported outcome measures, providing real-world evidence on feasibility, satisfaction, and perceived advantages and challenges.

This study aimed to prospectively evaluate a structured teleconsultation model for patients with primary headache using two standardized teleconsultations and validated patient-reported outcome measures. The primary objective was to assess feasibility and patient satisfaction, while secondary objectives included examining perceived benefits, identifying potential barriers, and monitoring changes in patient experience over time.

## Methods

### Study design

This was a prospective, single-center study conducted at the Headache Clinic of the University Hospital “Ospedali Riuniti” in Foggia (Italy). The study was carried out between January 2025 and July 2025. All patients underwent two scheduled teleconsultations as part of their routine clinical follow-up.

### Participants

A total of 45 patients with a diagnosis of primary headache [migraine, tension-type headache, or other primary forms according to the International Classification of headache disorder 3rd edition (ICHD-3)] (10) were consecutively enrolled. Inclusion criteria were: age ≥18 years, a confirmed diagnosis of primary headache according to the ICHD-3, at least one prior in-person neurological visit at our clinic, and the ability to use basic digital tools or access caregiver support. Exclusion criteria were: secondary headache disorders; cognitive impairment or severe psychiatric conditions that could interfere with participation or reliable questionnaire completion; inability to use or access the telemedicine platform due to lack of a suitable digital device, internet connection, or adequate digital literacy; insufficient comprehension of the Italian language; and refusal or inability to provide informed consent.

### Ethical considerations

All participants provided informed consent before participating. This study involved routine teleconsultation procedures already authorized and implemented within the hospital’s digital system; therefore, additional formal approval from the ethics committee was not required., in accordance with Italian regulations on observational studies conducted within standard clinical practice.

### Telemedicine procedure

Each patient underwent two teleconsultations: a baseline visit (T1) and a follow-up visit (T2), conducted every 4–8 weeks. Consultations were performed using the Promoting eHealth in Cross-Border Area by Stimulating Economy (PHASE) platform, a secure, GDPR-compliant digital environment that supports encrypted video consultations, document exchange, and protected data storage. No formal technical support service was available during the teleconsultations. However, all patients received basic instructions during a prior in-person visit, and the platform’s intuitive interface allowed autonomous access or caregiver assistance when needed.

### Outcomes and assessment tools

After each teleconsultation (T1 and T2), patients completed a 20-item anonymous online questionnaire designed to evaluate their telemedicine experience. The instrument, previously used in healthcare settings, assessed multiple domains, including technical aspects (e.g., connection quality, platform usability, and document exchange), physician–patient communication, perceived time and cost savings, overall patient satisfaction, and willingness to repeat a teleconsultation.

Most items were rated on a 7-point Likert scale, while additional binary and categorical questions collected demographic and contextual information (e.g., employment status, caregiver support, and distance from the hospital). The questionnaire was self-administered immediately after each teleconsultation. Internal reliability was high (Cronbach’s *α* = 0.92), indicating excellent internal consistency.

### Statistical analysis

Responses were analyzed at both T1 and T2. Descriptive statistics were reported as means and standard deviations. The Wilcoxon signed-rank test was used for T1–T2 comparisons. Correlations were assessed using Spearman’s coefficient. Subgroup analyses were conducted based on age, employment status, and caregiver presence. Effect sizes were calculated using Cohen’s *d* after data normalization.

## Results

All 45 patients completed the two-phase telemedicine protocol (Table 1). The majority lived more than 3 km from the hospital, with eight patients living over 100 km away. Sixty-nine percent were employed, and 24% required caregiver support during teleconsultations.

**Table 1:** Baseline characteristics of the study population (*N* = 45).

Demographic variables	Value category	Number of responses	%
Age	≤40 years	14	31.1%
	>40 years	31	68.9%
Home–to-hospital distance	≤50 km	30	66.7%
	>50 km	15	33.3%
Home-to-hospital travel time	<1 h	32	71.1%
	≥1 h	13	28.9%
Number of annual hospital visits	≤5	39	87.0%
	>5	6	13.0%
Patients engaged in employment	Yes	31	69.0%
	No	14	31.0%
Patients assisted by a caregiver	Yes	11	24.0%
	No	34	76.0%

Demographic and clinical features of enrolled patients. Data are expressed as a number (percentage). Percentages are rounded.

**Figure 1:**
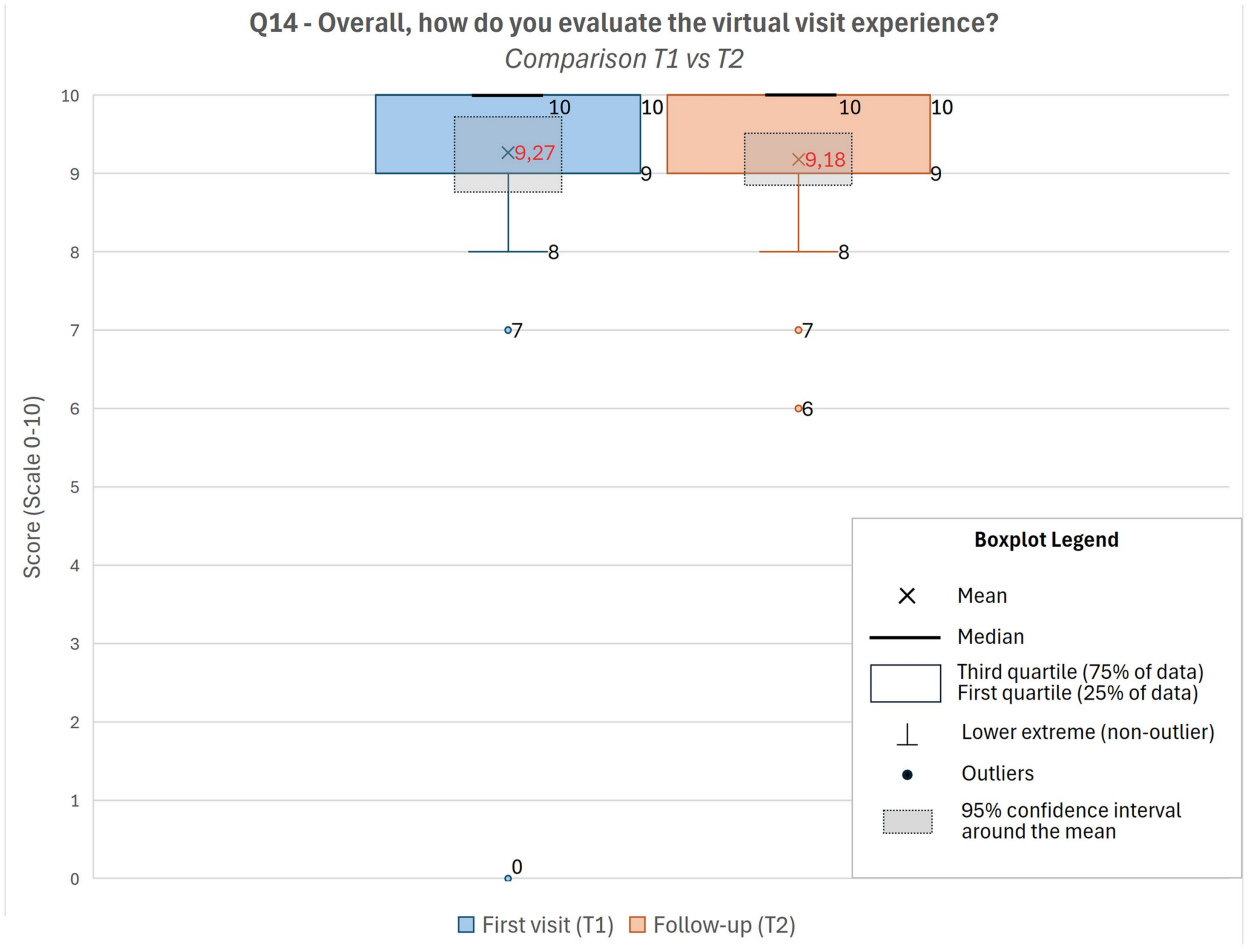
Overall satisfaction with teleconsultations at T1 and T2. Boxplots show patient ratings of overall satisfaction with virtual visits (Q14) at the first teleconsultation (T1) and follow-up (T2). Scores remained consistently high, with mean values above 9/10 at both time points.

**Figure 2:**
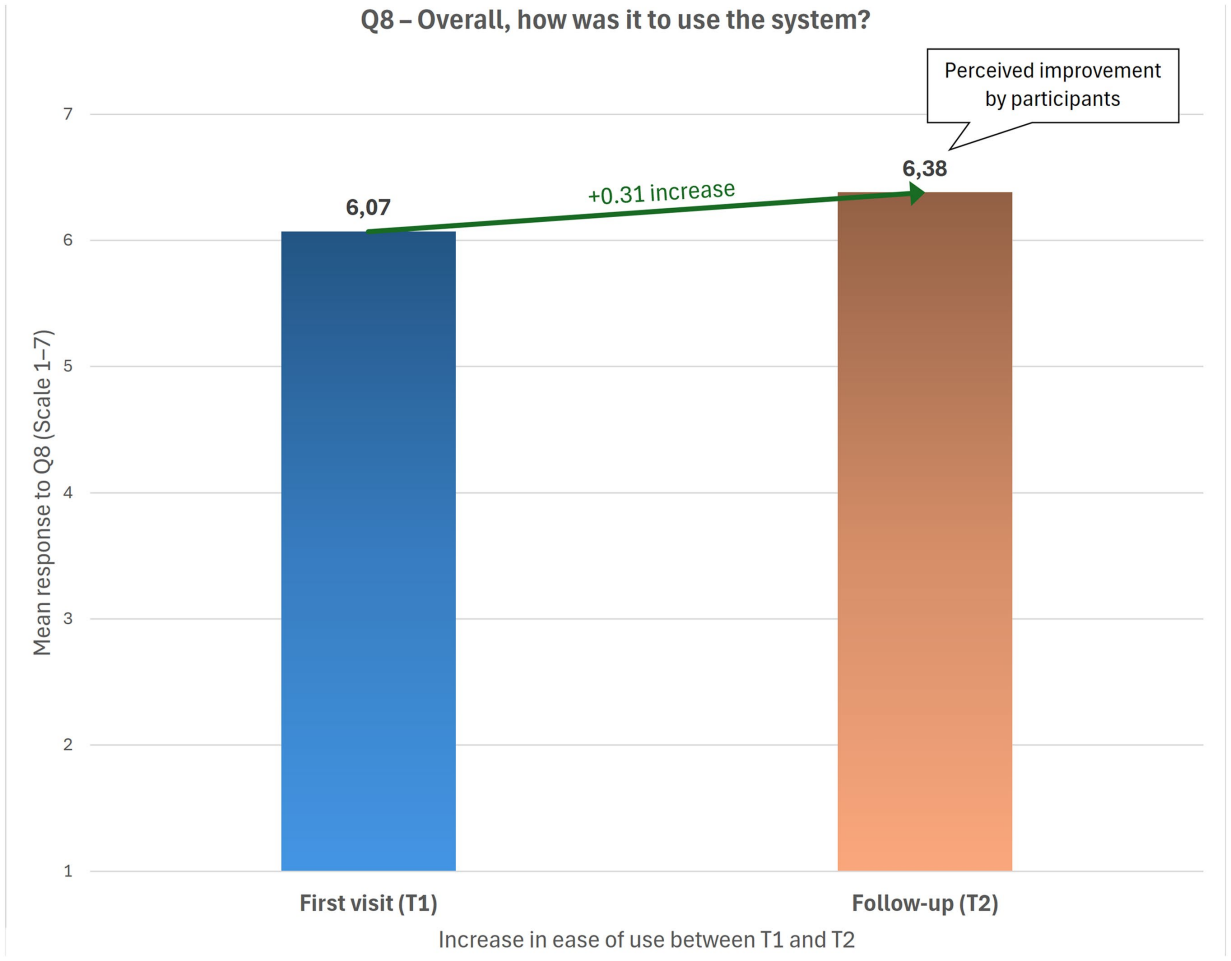
Ease of use of the telemedicine system at T1 and T2. Bar chart illustrates patient ratings of system usability (Q8) at the first teleconsultation (T1) and follow-up (T2). A tendency toward improvement was observed, suggesting increasing familiarity with the platform.

Overall satisfaction was very high from the first visit, with mean scores above 9/10 at both T1 and T2, and more than 90% of patients reported a positive experience (Figure 1). Ease of use reached an average of 6/7, with a statistically significant improvement from T1 to T2. Patients also reported progressive improvements in the perceived adequacy of consultation duration, document exchange, and economic and logistical benefits. More than 85% acknowledged tangible savings in both time and costs (Figure 2).

Wilcoxon tests confirmed significant improvements between T1 and T2 in platform usability, adequacy of instructions, document exchange, and consultation duration, as well as in the overall evaluation of the virtual visit, perceived economic savings, time savings, perceived benefits of telemedicine, and interest in undergoing further teleconsultations (*p* = 0.021). No significant differences were found for physician–patient communication and for the perceived benefit of avoiding hospital access, both of which were already positively rated at baseline.

**Figure 3:**
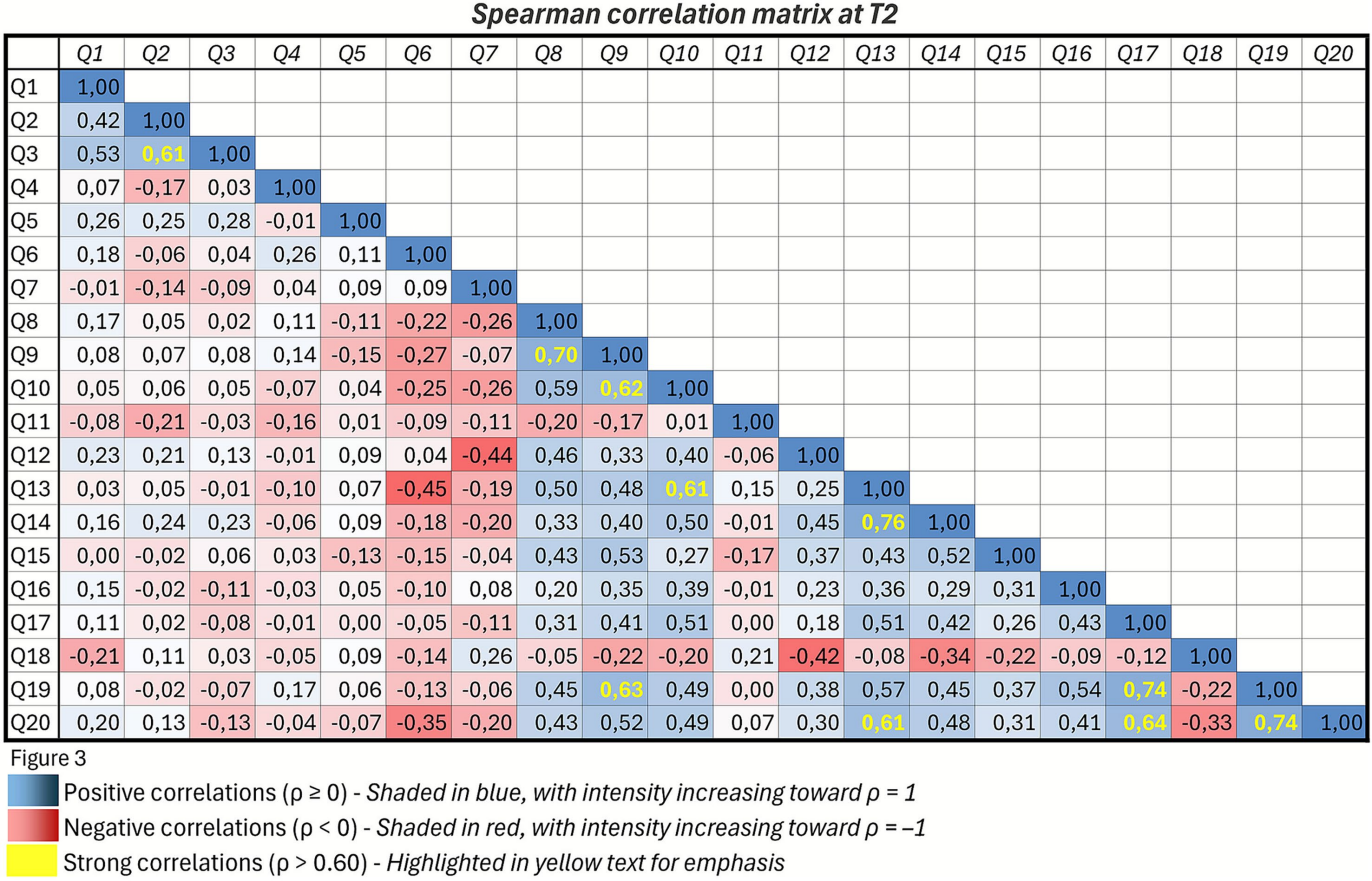
Spearman’s correlation matrix for questionnaire items at T2. Each cell represents the correlation coefficient (*ρ*) between a pair of items. The diagonal contains self-correlations (*ρ* = 1). Only the lower triangle of the matrix is displayed to avoid redundancy. Conditional formatting highlights positive correlations (*ρ* ≥ 0) in shades of blue and negative correlations (*ρ* < 0) in shades of red. Correlations greater than 0.60 are additionally marked in yellow to indicate strong associations.

Spearman’s correlations showed strong positive associations between overall satisfaction (Q14) and perceived consultation duration (Q13) (*ρ* = 0.76), as well as with willingness to repeat the teleconsultation (Q20) (*ρ* = 0.61) (Figure 3). Time savings (Q17) are strongly correlated with avoidance of hospital access (Q19) (*ρ* = 0.74) and with willingness to repeat the teleconsultation (Q20) (*ρ* = 0.64). A strong correlation was also observed between participants’ overall experience with the telemedicine system (Q8) and their evaluation of the adequacy of the instructions provided before the consultation (Q9) (*ρ* = 0.70) (Figure 3).

Subgroup analyses revealed that patients ≤40 years reported lower ratings at T1 for physician–patient communication, economic savings, and time savings; however, these differences were no longer significant at T2. Employed patients initially reported higher ease of use, while unemployed patients showed greater improvement between T1 and T2. Patients who received caregiver support reported higher satisfaction at T1, which slightly decreased at T2, whereas those without caregiver support showed a steady increase in satisfaction over time.

Effect-size analysis confirmed a minimal difference in overall satisfaction between T1 and T2 (Cohen’s *d* = 0.07), consistent with a ceiling effect due to the high baseline ratings. Small-to-moderate effect sizes were observed for ease of use, document exchange, and willingness to repeat the teleconsultation (*d* = 0.24–0.27).

## Discussion

The results confirm that telemedicine represents a feasible, safe, and effective modality for neurological follow-up in patients with primary headache, as evidenced by a high rate of completed visits and minimal technical difficulties. These findings are consistent with recent literature reporting comparable completion rates and strong patient acceptance, indicating that telemedicine can ensure continuity of care and achieve clinical outcomes similar to in-person consultations, while reducing waiting times and improving continuity of assistance (11, 12). The study protocol included two structured teleconsultations and a validated satisfaction questionnaire, providing additional methodological rigor and reproducibility.

Satisfaction levels were notably very high following the first teleconsultation and remained stable at follow-up, in line with previous studies reporting comparable or even higher satisfaction with telemedicine compared with in-person visits (13). Patient satisfaction in our cohort was high and showed a significant improvement between the first and second teleconsultation, suggesting increasing familiarity and confidence with the digital platform. This finding is consistent with prior research indicating that telemedicine enhances accessibility, reduces travel time, and preserves the quality of doctor–patient communication (14).

Beyond telemedicine, recent literature has highlighted the role of other digital interventions in neurological and headache care, such as remote digital training and tele-education programs, which support patient empowerment, self-management, and treatment adherence. In particular, telecoaching models have demonstrated promise in guiding patients through digital tools and maintaining engagement over time, especially among individuals with low digital literacy. Integrating these approaches with structured teleconsultations may further enhance continuity of care and improve the patient experience in virtual headache management (15, 16).

Platform usability improved significantly between visits, suggesting progressive familiarization with the system. Patients also reported substantial time and cost savings, underscoring the logistical and economic benefits of teleconsultations. This is particularly relevant in the Italian context, where many patients must travel long distances to access specialized headache centers.

Physician–patient communication was perceived as comparable to in-person interaction, confirming that telemedicine can preserve relational quality when adequate time and empathetic engagement are ensured. Correlation analyses further supported this finding, showing significant associations between satisfaction, perceived consultation duration, and willingness to repeat teleconsultations.

Subgroup analyses indicated that younger patients and unemployed individuals showed greater improvements over follow-up, as did those without caregiver support, suggesting that initial barriers related to digital literacy may be progressively reduced over time.

Despite these positive results, this study has several limitations, including the small sample size, single-center design, anonymity of responses—which prevented individual longitudinal analyses—and the absence of direct clinical outcomes such as headache frequency or intensity. Moreover, the inability to perform a full neurological examination remains an intrinsic limitation of telemedicine.

Nevertheless, this study provides real-world evidence supporting the use of telemedicine in headache care. By confirming high satisfaction levels, logistical advantages, and stable perceptions over time, our findings reinforce the rationale for integrating telemedicine into follow-up pathways. Although this study was not specifically designed to evaluate long-term clinical outcomes, our observations suggest that telemedicine follow-up may support treatment adherence and reduce unnecessary emergency department visits. These results are consistent with previous studies reporting that structured teleconsultations improve continuity of care, facilitate long-term monitoring in patients with chronic headache, and ensure timely access to follow-up when needed (17–20).

Overall, our findings support the hypothesis that telemedicine can be implemented as a stable component of headache-care pathways, maintaining effectiveness and quality comparable to in-person follow-up. Future research should explore hybrid models that combine telemedicine with selected in-person visits and develop digital literacy support strategies to improve accessibility for patients with limited technological skills.

## Conclusion

A telemedicine protocol for headache follow-up proved feasible, well accepted, and associated with high levels of patient satisfaction and perceived benefits. Teleconsultations optimized resource utilization while maintaining the quality of physician–patient communication, despite the inherent limitation of not performing a full physical examination. Multicenter randomized studies with larger samples and clinical endpoints are needed to consolidate these findings and define best practices for hybrid care models.

## Data Availability

The original contributions presented in the study are included in the article/supplementary material, further inquiries can be directed to the corresponding author.
